# Predicting progression from SeLECTS with SWAS to EE-SWAS: risk factor identification and model development

**DOI:** 10.3389/fnhum.2025.1641421

**Published:** 2026-01-15

**Authors:** Qiao Hu, Yuanyuan Luo, Yu Deng, Lingling Xie, Jiannan Ma, Siqi Hong, Ping Yuan, Li Jiang

**Affiliations:** Department of Neurology, Children’s Hospital of Chongqing Medical University, National Clinical Research Center for Children and Adolesents’ Health and Diseases, Ministry of Education Key Laboratory of Child Development and Disorders, Chongqing Key Laboratory of Child Neurodevelopment and Cognitive Disorders, Chongqing, China

**Keywords:** SeLECTS, EE-SWAS, prediction model, SWAS, risk factor

## Abstract

**Introduction:**

This study sought to identify early risk factors and develop a predictive model for progression from self-limited epilepsy with centrotemporal spikes (SeLECTS) accompanied by spike-and-wave activation in sleep (SWAS) to epileptic encephalopathy with SWAS (EE-SWAS), aiming to facilitate early clinical intervention.

**Methods:**

From a pediatric cohort with spike-and-wave index >50%, we analyzed 77 SeLECTS patients (33 progressed to EE-SWAS, 36 remained stable over ≥2 years of follow-up). Baseline clinical and EEG features were comprehensively evaluated. Multivariate logistic regression identified independent predictors of cognitive regression, which were incorporated into a nomogram-based predictive model. Model performance was assessed using the C-index in both derivation and external validation cohorts.

**Results:**

Prolonged spike-and-wave clusters, high-amplitude spikes with secondary generalization, and younger age at first seizure emerged as independent predictors of EE-SWAS progression. The nomogram model demonstrated high discriminative ability, with a C-index of 0.932 in the derivation cohort and 0.934 in external validation.

**Conclusion:**

This study provides the first validated tool for early risk stratification in SWAS-associated SeLECTS, enabling clinicians to anticipate EE-SWAS progression and optimize therapeutic strategies. The model’s robustness supports its potential utility in clinical decision-making to mitigate cognitive decline.

## Introduction

Self-limited epilepsy with centrotemporal spikes (SeLECTS), formerly known as benign Rolandic epilepsy or benign childhood epilepsy with centrotemporal spikes, is the most common self-limited partial epilepsy syndrome, accounting for approximately 20% of epilepsy syndromes in children under 15 years of age (Specchio et al., 2022). SeLECTS is characterized by early school age of onset, centrotemporal electroencephalogram (EEG) spikes, and a self-limiting clinical course, all of which contribute to its recognized favorable prognosis (Specchio et al., 2022; Fejerman, 2009). While rare (∼4.6%), SeLECTS can progress to spike-and-wave activation in sleep (SWAS) with accompanying cognitive regression or stagnation, warranting reclassification as EE-SWAS—a condition carrying significant risk of lasting neurological sequelae (Tovia et al., 2011).

Although not universal in SWAS, cognitive decline typically emerges after a variable latency period. While this interval’s exact duration requires further study, most cases demonstrate cognitive regression or stagnation within 2 years post-seizure onset (Specchio et al., 2022; Fejerman, 2009; Tovia et al., 2011; Halász and Szũcs, 2023; Ucar et al., 2022). Contrasting findings emerge from long-term follow-up studies. Bebek et al. reported no cognitive regression or stagnation in SWAS patients over a mean follow-up period of 14 years (Bebek et al., 2015). Similarly, Sibony and Kramer’s cohort study of 17 SeLECTS patients (mean follow-up: 5.5 years) found no cognitive or behavioral deterioration despite a mean SWI of 60% (Uliel-Sibony and Kramer, 2015). These observations suggest that SWAS may represent a benign EEG phenomenon in certain patients, potentially obviating the need for aggressive polypharmacy in such cases. Future studies should prioritize standardized EEG biomarkers and multidisciplinary approaches to bridge this translational gap.

Early diagnosis and treatment are critical in children with EE-SWAS to mitigate neurocognitive decline and prevent irreversible damage (Caraballo et al., 2014). While Massa et al. identified certain EEG features that might predict the progression from SeLECTS to EE-SWAS, these findings lack clinical consensus and practical applicability (Massa et al., 2001). Therefore, early identification of high-risk factors for cognitive regression or stagnation induced by continuous SWAS—alongside the development of a clinically actionable predictive model—remains a pressing priority.

In this study, we defined SWAS as the presence of spike-and-wave index (SWI) exceeding 50% of the sleep epoch, consistent with prior criteria (Arican et al., 2021; Gencpinar et al., 2016). Children with SeLECTS underwent serial EEG monitoring over a two-year follow-up period. Based on cognitive and EEG profiles, patients were stratified into two cohorts: (1) EE-SWAS and (2) SeLECTS with SWAS. Statistical analyses were conducted to delineate risk factors predisposing SeLECTS patients to EE-SWAS progression. Subsequently, a predictive model was developed to facilitate early therapeutic intervention and optimize long-term outcomes.

## Materials and methods

### Patient selection and protocol design

**Figure 1:**
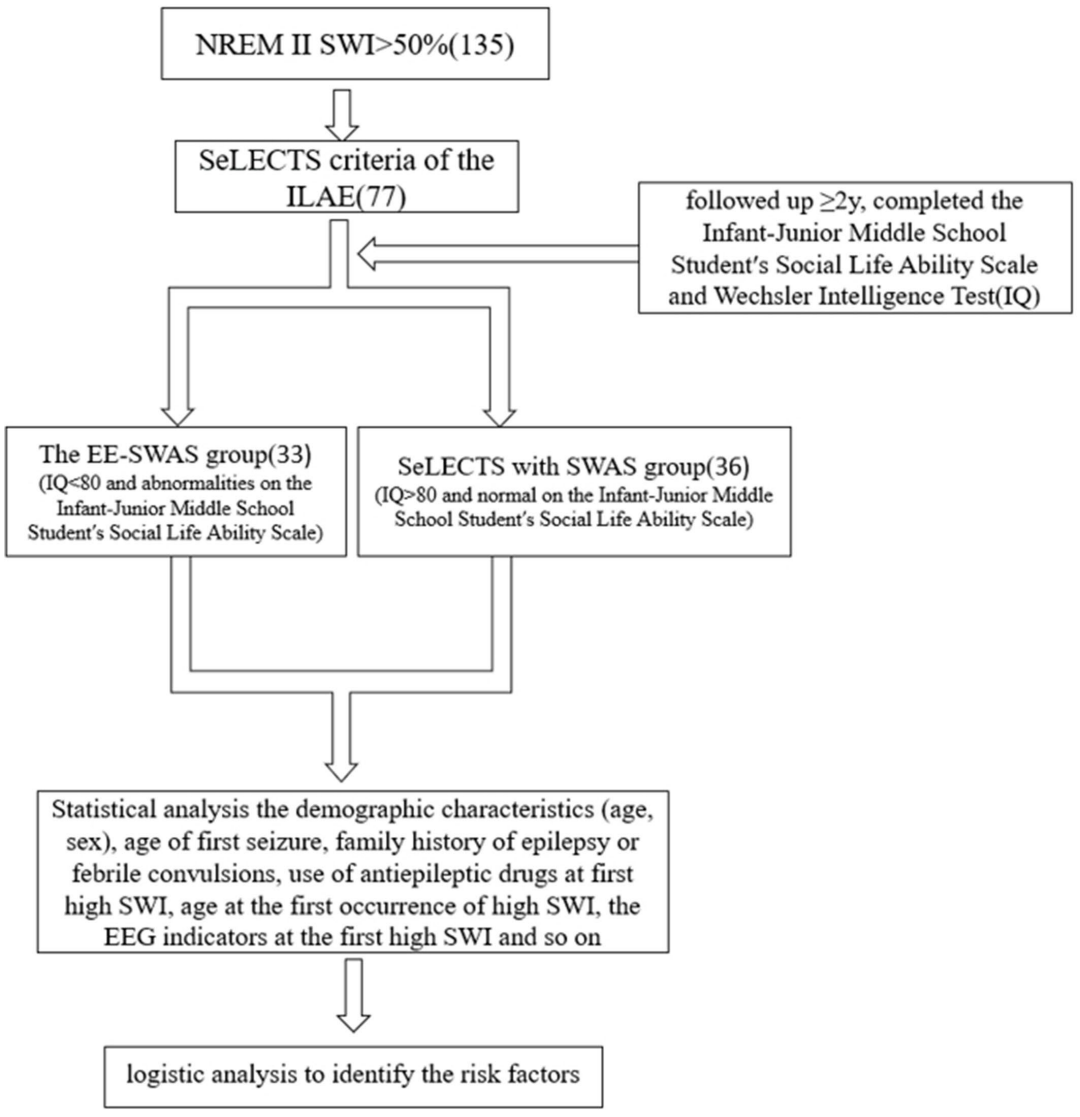
A flow chart of protocol design.

This study was conducted at a Level 3 Epilepsy Center within Children’s Hospital of Chongqing Medical University, China. We initially screened 135 children exhibiting SWI > 50% during nonrapid eye movement sleep stage (NREM) II sleep at our Liangjiang Campus. Based on ILAE SeLECTS criteria (clinical/MRI findings) (Specchio et al., 2022), 77 children were enrolled. All patients had normal cognition before SWAS onset on EEG. Upon SWAS detection, participants immediately underwent the Infant-Junior Middle School Student’s Social Life Ability Scale and Wechsler Intelligence Test. These assessments were repeated over the subsequent 2 years. Children displaying cognitive regression/stagnation—attributed to intense sleep-related epileptic activity—were classified as EE-SWAS (*n* = 33), while those with sustained normal cognition comprised the SeLECTS with SWAS group (*n* = 36). Eight patients were excluded due to loss to follow-up. For the modeling cohort, we collected: demographic data (age, sex), seizure onset age, family history of epilepsy/febrile convulsions, antiseizure medication use, seizure frequency at first SWAS detection, age at SWAS onset, and initial SWAS EEG parameters. A flow chart of this process is shown in Figure 1.

**Figure 2:**
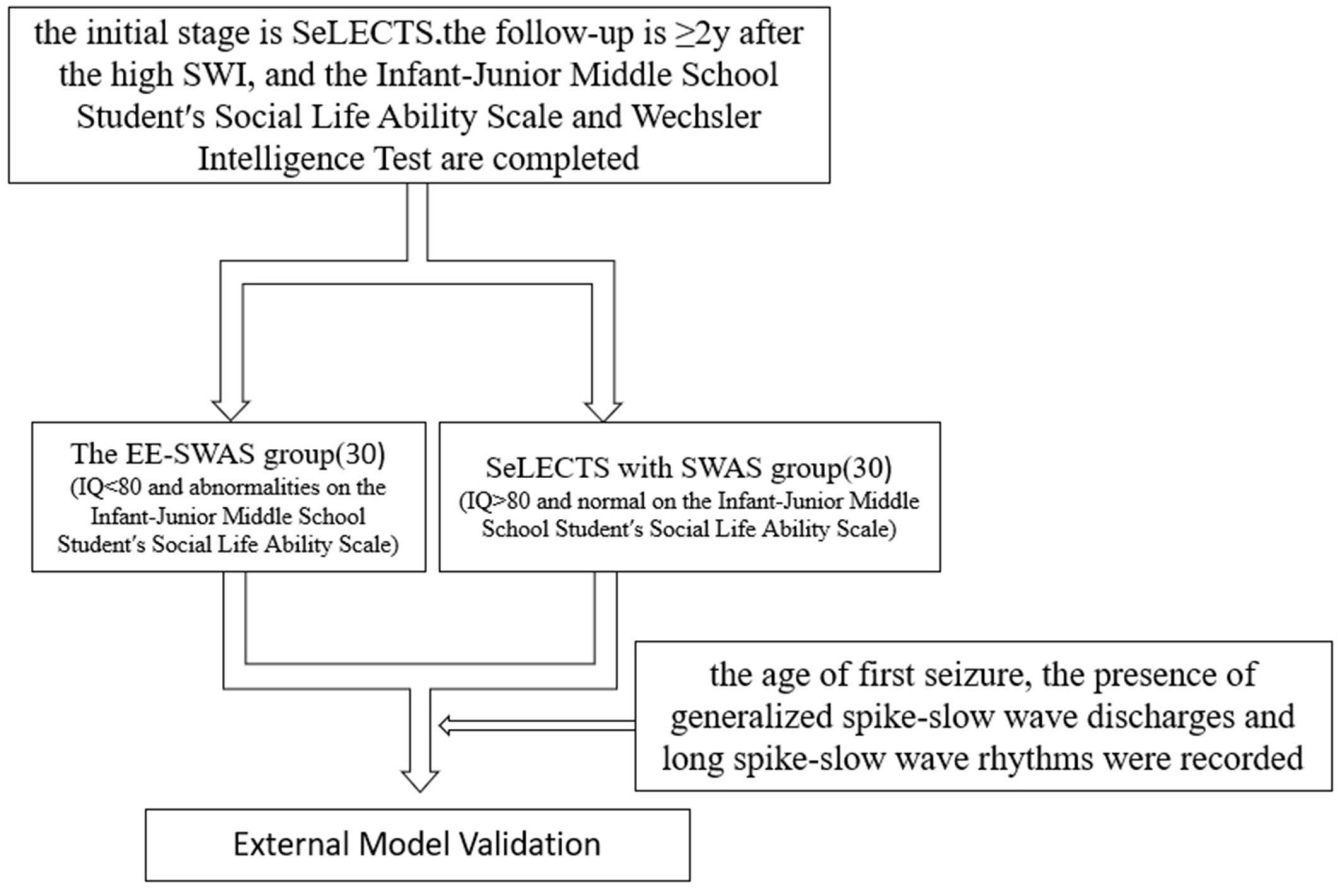
The workflow for cohort selection.

Using the model-identified risk factors, we established an external validation cohort (*n* = 60) following identical inclusion/exclusion criteria, comprising 30 EE-SWAS and 30 SeLECTS with SWAS patients from our Yuzhong Campus. In this cohort, only the age of first seizure, the high-amplitude SW with secondary generalization, and the long spike-and-wave clusters were recorded. See Figure 2 for cohort selection workflow.

This study was approved by the Ethics Committee of Children’s Hospital of Chongqing Medical University. Written informed consent was obtained from all participants and their guardians.

### EEG recording and analysis

EEGs were recorded using an international standard 10–20 system with a sampling frequency of 1,000 Hz (EEG-1200C, Nihon Kohden). The participants were deprived of sleep the night before the EEG examination. All patients were monitored by video-EEG for at least 4 h, and the EEGs during the waking period and stage NREM I/II/III sleep were recorded. EEG analysis was performed using the average or bipolar reference montage.

### EEG analysis

**Figure 3:**
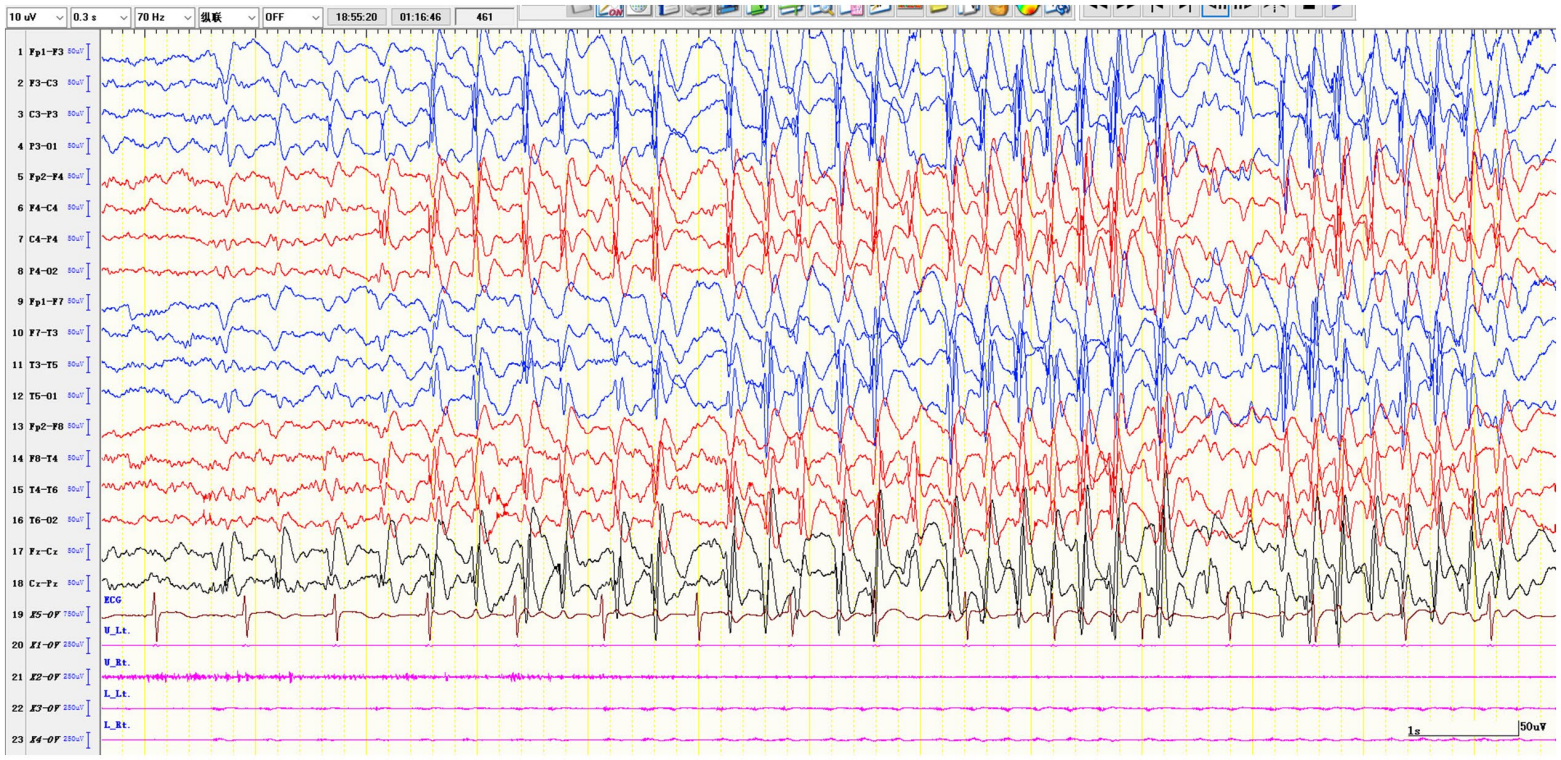
The high-amplitude SW with secondary generalization.

**Figure 4:**
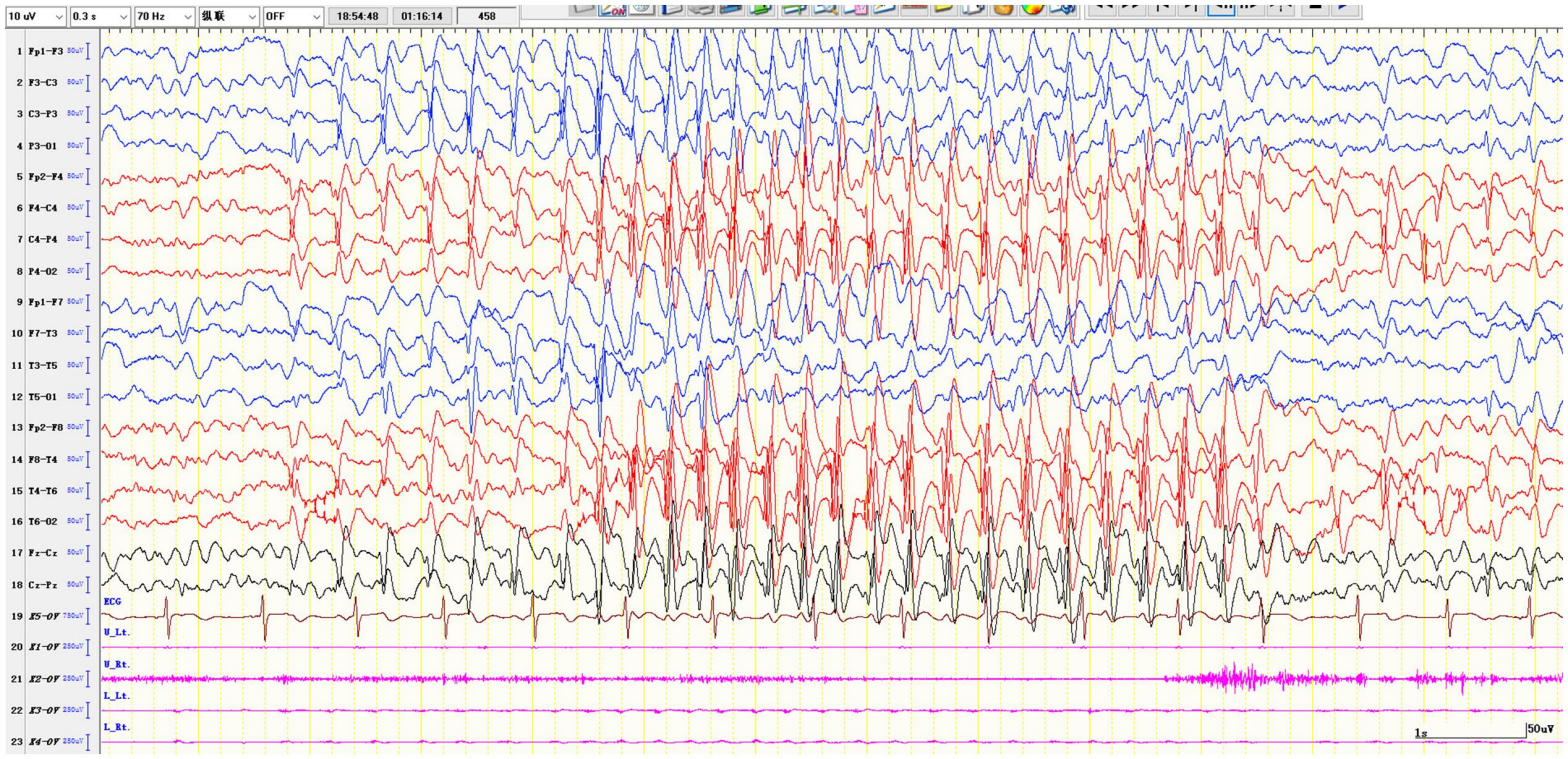
The long spike-and-wave clusters.

We analyzed eight EEG parameters in the cohort: (1) interictal centrotemporal discharge laterality (left/right/bilateral), (2) sleep SWI (spike-and-wave duration in first 10 min NREM II/600 s × 100%) (Uliel-Sibony and Kramer, 2015; Ji et al., 2023), (3) wake discharge frequency (counts/5 min), (4) non-dipole spikes (frontotemporal dipole incidence <80%) (Gregory and Wong, 1992), (5) multifocal discharges (≥2 non-Rolandic foci) (Massa et al., 2001), (6) intermittent focal slow waves, (7) high-amplitude SW with secondary generalization (3 Hz absence-like, 1–5 s) (Massa et al., 2001; Nicolai et al., 2007; Aeby et al., 2021) (Figure 3), and (8) the long spike-and-wave clusters (≥6 s) (Massa et al., 2001; Nicolai et al., 2007; van Klink et al., 2016; Figure 4), with only parameters 7–8 assessed in the validation cohort, all independently reviewed by two board-certified epileptologists (Q. H., P. Y.) with >10 years’ experience.

### Statistics

Statistical analyses were performed using SPSS 26.0 (IBM Corp.). Group comparisons (EE-SWAS vs. SeLECTS with SWAS) employed χ^2^ tests, Fisher’s exact tests, *t*-tests (normal distribution), and Mann–Whitney U tests (non-normal distribution). Multivariate logistic regression identified risk factors for EE-SWAS progression, preceded by multicollinearity assessment (tolerance >0.1, VIF < 10, condition index <30). Significant predictors (*p* < 0.05) were incorporated into a nomogram, with model performance evaluated via ROC curve analysis (AUC, sensitivity, specificity). External validation was subsequently conducted on an independent cohort.

## Results

### Demographic and clinical data

A total of 69 children were included in the model cohort. Notably, the EE-SWAS group exhibited significantly earlier seizure onset (5.60 ± 1.67 years) compared to the SeLECTS with SWAS group (7.39 ± 1.71 years; *p* < 0.001). Febrile convulsion history (*p* = 0.024) and epilepsy family history (*p* = 0.008) also showed statistically significant intergroup differences. For comprehensive demographic and clinical characteristics, refer to Table 1.

**Table 1:** Clinical data of the EE-SWAS group and the SeLECTS with SWAS group.

Clinical data	EE-SWAS (*n* = 33)	SeLECTS with SWAS (*n* = 36)	χ^2^/t/Z	*p*-value
Age at first seizure	5.60 ± 1.67	7.39 ± 1.71	−4.397	0.000
Age at first occurrence of SWAS	7.92 ± 1.90	8.78 ± 2.11	−1.767	0.082
Sex			3.152	0.076
Female	15(45.45)	24 (66.67)		
Male	18 (54.55)	12 (33.33)		
Number of attacks			13.644	0.008
2	15(45.45)	14 (38.89)		
3	4 (12.12)	17 (47.22)		
4	6 (18.18)	2 (5.56)		
5	4 (12.12)	3 (8.33)		
6	4 (12.12)	0 (0)		
Number of antiepileptic drugs			2.550	0.148
1	21 (63.64)	16 (44.44)		
2	12 (36.36)	20 (55.56)		
History of febrile seizure/family history of epilepsy			5.708	0.024
None	26 (78.79)	35 (97.22)		
Yes	7 (21.21)	1 (2.78)		

### EEG characteristics

EE-SWAS group showed significantly higher prevalence of high-amplitude SW with secondary generalization (60.61% vs. 2.78%, *p* < 0.001) and prolonged spike-and-wave clusters (66.67% vs. 5.56%, *p* < 0.001), along with greater median SWI (0.78 vs. 0.62) and bilateral interictal discharges (75.76% vs. 38.89%) compared to SeLECTS+SWAS group, with additional significant differences in nondipole spikes and discharge frequency (all *p* < 0.05) but not in multifocal discharges or slow waves, while pre-SWAS ictal EEG frequency was also higher in EE-SWAS (*p* = 0.014), all based on first SWAS detection EEG data (see Table 2).

**Table 2:** EEG data of the EE-SWAS group and the SeLECTS with SWAS group.

EEG data	EE-SWAS (*n* = 33)	SeLECTS with SWAS (*n* = 36)	χ^2^/t/Z	*P*-value
Discharge frequency during waking period (times/min)	20.00 (30.50)	4.20 (13.60)	−3.715	0.000
SWI	0.78 (0.21)	0.62 (0.20)	−4.682	0.000
Ictal EEG			6.060	0.014
No	24 (72.73)	34 (94.44)		
Yes	9 (27.27)	2 (5.56)		
Centrotemporal discharges			9.812	0.007
Right	5 (15.15)	11 (30.56)		
Left	3 (9.09)	11 (30.56)		
Bilateral	25 (75.76)	14 (38.89)		
Nondipole spike wave			7.001	0.008
No	8 (24.24)	20 (55.56)		
Yes	25 (75.76)	16 (44.44)		
Multiple asynchronous discharges			0.047	1.000
No	29 (87.88)	31 (86.11)		
Yes	4 (12.12)	5 (13.89)		
High-amplitude SW with secondary generalization			27.195	0.000
No	13 (39.39)	35 (97.22)		
Yes	20 (60.61)	1 (2.78)		
Intermittent slow wave lesions			2.678	0.140
No	27 (81.82)	34 (94.44)		
Yes	6 (18.18)	2 (5.56)		
Long spike–wave clusters			28.345	0.000
No	11 (33.33)	34 (94.44)		
Yes	22 (66.67)	2 (5.56)		

### Multivariate regression analysis

Multivariate logistic analysis of the clinical and EEG data revealed that long spike-and-wave clusters, high-amplitude SW with secondary generalization, and young age of first seizure were risk factors for the progression of SeLECTS with SWAS into EE-SWAS. Detailed data are shown in Table 3.

**Table 3:** Multivariate logistic analysis results.

Variable	*B*	SE	OR	95% CI	*p*-value
Long spike–wave clusters	2.101	1.027	8.171	1.091 ~ 61.197	0.041
Age of first seizure	−0.847	0.280	0.429	0.248 ~ 0.742	0.002
High-amplitude SW with secondary generalization	3.976	1.604	53.314	2.297 ~ 1237.599	0.013
Constant	3.952	1.718	52.023		0.021

### Construction and validation of the predictive model

**Figure 5:**
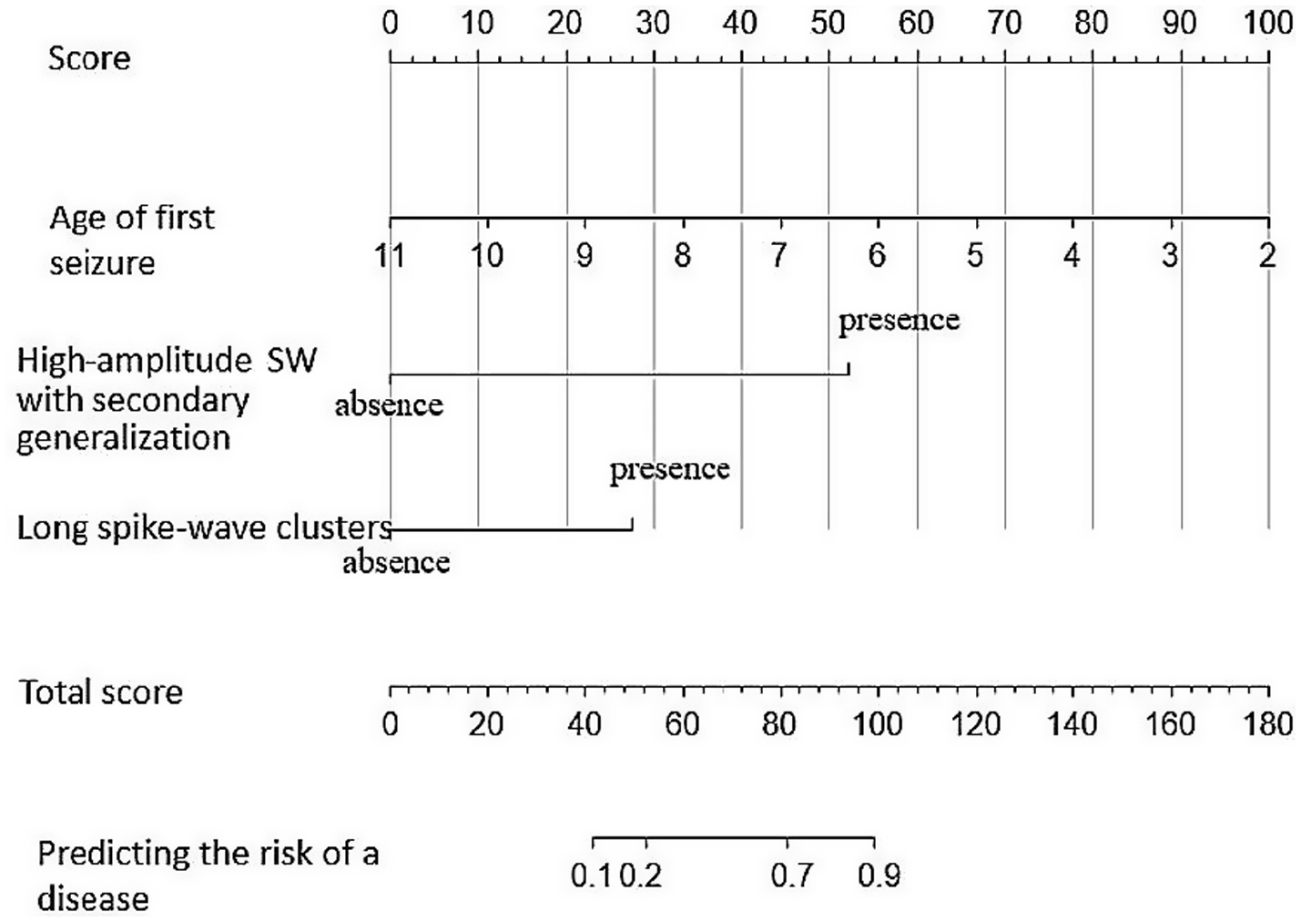
The predictive model with nomogram.

Individual risk factor scores were derived from the nomogram, with the cumulative score predicting the probability of EE-SWAS progression in SeLECTS patients with SWAS (Figure 5).

**Figure 6:**
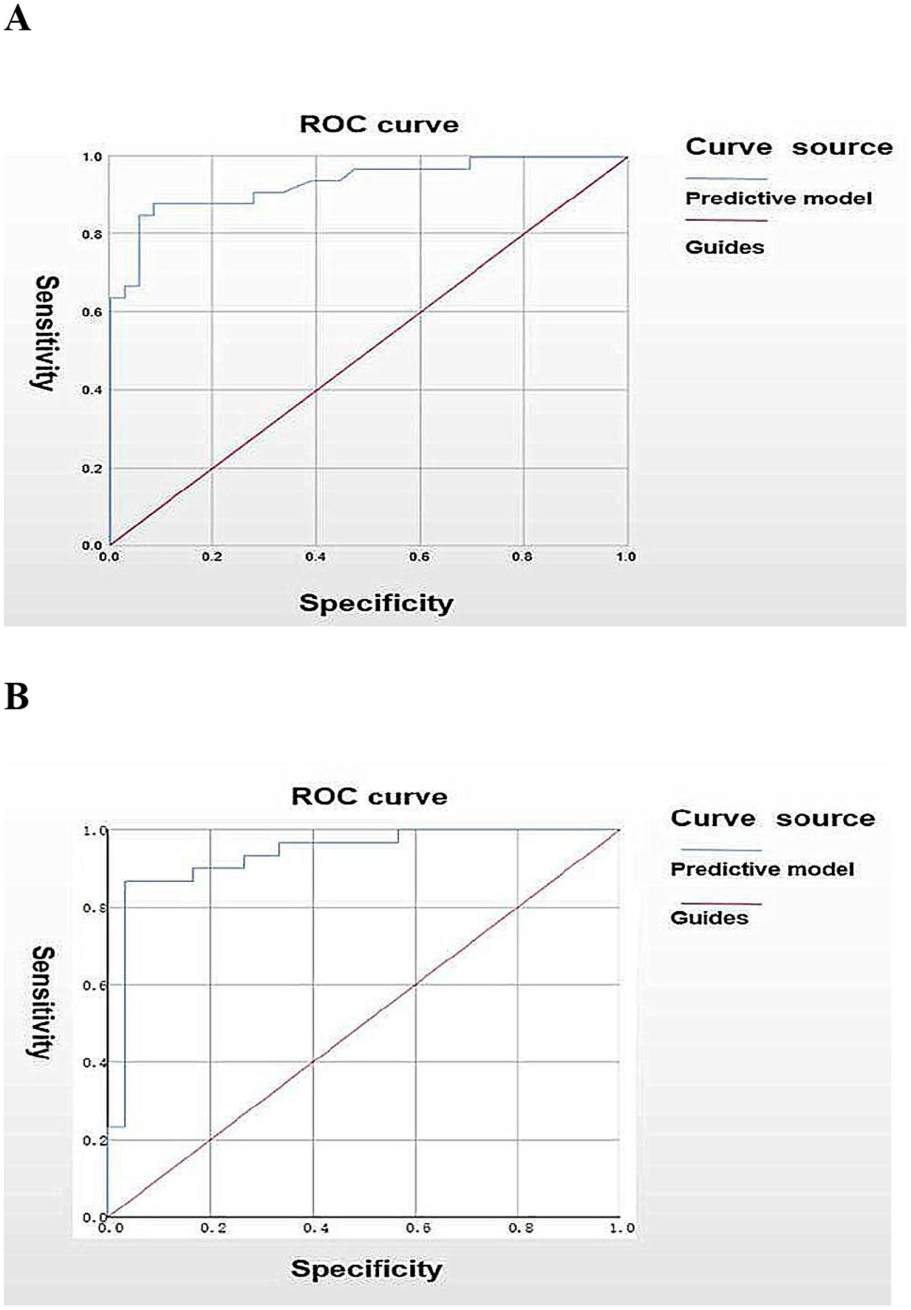
Receiver operating characteristic (ROC) curve of the prediction model. **(A)** ROC curve of the prediction model for the probability of disease in the EE-SWAS group. **(B)** ROC curve of the prediction model for the probability of disease in the validation group.

The predictive model demonstrated excellent discriminative ability, with a C-index of 0.932 (95% CI: 0.872–0.992, *p* < 0.001) in the derivation cohort and 0.934 (95% CI: 0.868–1.000, *p* < 0.001) in the external validation cohort using three key predictors (younger seizure onset age, the long spike-and-wave clusters, and high-amplitude SW with secondary generalization), confirming robust performance in stratifying progression risk from SeLECTS with SWAS to EE-SWAS (Figures 6A,B).

## Discussion

SeLECTS, the most prevalent self-limited focal epilepsy syndrome, carries a 7% risk of devastating neurocognitive decline (Kramer, 2008). Early intervention targeting encephalopathic EEG patterns may prevent irreversible damage (Fejerman, 2009), underscoring the need to identify progression predictors. Our study reveals the long spike-and-wave clusters (≥6 s), high-amplitude SW with secondary generalization, and younger seizure onset age as key risk factors for EE-SWAS progression. We developed the first validated nomogram (derivation C-index: 0.932; validation C-index: 0.934) to quantify this risk, addressing a critical gap in early prognostication.

### Clinical high-risk factors: age at first seizure

Prior studies identified various risk factors for EE-SWAS progression: early seizure onset and increased frequency (Bebek et al., 2015; Desprairies et al., 2018), dysarthria/somatosensory auras (Porat Rein et al., 2021), multiple seizure types (Caraballo et al., 2019), and new seizure emergence (Desprairies et al., 2018). While these studies reported diverse factors, we confirmed younger seizure onset age as paramount-consistent with Fejerman/Posar (Fejerman, 2009; Posar and Visconti, 2024) and You et al.’s finding that SeLECTS onset <3 years predicts severe progression (You et al., 2006). Our data showed significantly earlier onset in EE-SWAS (5.60 ± 1.67 years) versus SeLECTS+SWAS (7.39 ± 1.71 years). The mechanism linking early seizure onset to atypical neurodevelopment (particularly cognitive regression/stagnation) remains unclear. Neuroimaging evidence suggests subtle structural remodeling occurs in both epileptogenic zones and distal regions of SeLECTS brains (Garcia-Ramos et al., 2015; Pardoe et al., 2013), with progressive changes observed in cognitively impaired patients (Garcia-Ramos et al., 2015; Pardoe et al., 2013; Ciumas et al., 2017). This implies epileptogenesis may disrupt normal developmental trajectories through cumulative interference with neuronal network maturation (Maltoni et al., 2016; Bourel-Ponchel et al., 2019). Earlier seizure onset likely extends this disruptive exposure window, amplifying neurodevelopmental risks.

While age of onset, febrile seizure history, epilepsy family history, and seizure frequency showed univariate significance, only age retained predictive value in multivariate analysis. The familial patterns suggest genetic predisposition–GRIN2A mutations (affecting synaptic GluN2A) (Gong et al., 2021) and CNKSR2 variants (Higa et al., 2021) are established EE-SWAS contributors, with additional genes (KCNA2, SLC9A6, HIVEP2, RARS2) implicated in developmental/epileptic encephalopathies with SWAS (Gong et al., 2021; Gong et al., 2020). Limited genetic data in our cohort (3 exome-sequenced cases: 1 negative, 1 SCN1A/SCN8A heterozygous, 1 PTEN heterozygous) preclude definitive conclusions.

### High-amplitude SW with secondary generalization and long spike-and-wave clusters

Beyond seizure onset age, SeLECTS interictal EEGs demonstrate atypical patterns—multifocal discharges, high-amplitude SW with secondary generalization, focal slow waves, and prolonged spike-and-wave clusters (Massa et al., 2001; van Klink et al., 2016). Crucially, these patterns (not discharge frequency) better predict neurocognitive outcomes (Bourel-Ponchel et al., 2019; Metz-Lutz and Filippini, 2006), consistent with Gencpinar et al.’s finding that SWI (>85% vs. 50–85%) lacked clinical correlation (Gencpinar et al., 2016). Our data confirm that high-amplitude SW with secondary generalization and prolonged clusters—not SWI—independently predict EE-SWAS progression.

First described in 1976 (Bernardina and Beghini, 1976), high-amplitude SW with secondary generalization which also described as 3 Hz generalized spike-and-waves (lasting 1–5 s during NREM I/II sleep; Gelisse et al., 1999) and prolonged spike-and-wave clusters (≥6 s across sleep stages; Massa et al., 2001; Berroya et al., 2005) are hallmark EEG patterns in SeLECTS/EE-SWAS. While their cognitive impairment mechanism remains unclear, synaptic homeostasis disruption during sleep is hypothesized—evidenced by absent sleep slow-wave activity (SWA) changes in active EE-SWAS, with post-remission SWA normalization (Rubboli et al., 2019; Rubboli et al., 2023). This suggests spike-induced interference with synaptic pruning, potentially explaining EE-SWAS neuropsychological deficits and guiding future research.

### Prediction model

While the mechanisms linking our three identified risk factors (early seizure onset, high-amplitude SW with secondary generalization, and prolonged spike-and-wave clusters) to cognitive regression or stagnation remain unclear, this first externally validated prediction model for EE-SWAS progression in SeLECTS patients provides clinically actionable insights. Statistically robust calculations confirm these factors enable early risk stratification, allowing targeted intensive monitoring and preemptive therapy for high-risk cases to mitigate neurocognitive deterioration (van den Munckhof et al., 2015; van den Munckhof et al., 2018).

Managing cognitive impairment in EE-SWAS remains challenging, with no established optimal treatment (Sánchez Fernández et al., 2014). While conventional antiseizure drugs show limited efficacy [49% improvement rate (van den Munckhof et al., 2020)], benzodiazepines (68%) and glucocorticoids (81%) demonstrate better outcomes. However, their use lacks standardized protocols (Sánchez Fernández et al., 2012; Veggiotti et al., 2012), and side effects (sedation, metabolic disturbances, infection risks) warrant caution. Our prediction model addresses this dilemma: high-risk cases may justify aggressive therapy, whereas low-risk patients—despite evident SWAS—should avoid unnecessary steroid/benzodiazepine exposure, as supported by Bebek/Posar’s findings (Bebek et al., 2015; Posar and Visconti, 2024). Crucially, treatment goals should prioritize cognitive impact over purely achieving EEG normalization.

This single-center cohort study has inherent limitations, including potential selection bias and restricted sample size affecting model calibration. While multicenter validation would enhance generalizability, our model establishes foundational predictors for cognitive regression or stagnation in SeLECTS with SWAS. These factors warrant targeted investigation through hypothesis-driven studies and longitudinal validation in larger cohorts.

## Conclusion

In summary, prolonged spike-and-wave clusters, high-amplitude SW with secondary generalization, and early seizure onset independently predict cognitive regression or stagnation in SeLECTS with SWAS progressing to EE-SWAS. We developed a predictive model to aid clinicians in early risk stratification and therapeutic decision-making.

## Data Availability

The original contributions presented in the study are included in the article/supplementary material, further inquiries can be directed to the corresponding author.
